# Reproductive Markers of Testicular Function and Size During Puberty in Boys With and Without a History of Cryptorchidism

**DOI:** 10.1210/clinem/dgac520

**Published:** 2022-09-08

**Authors:** Wiwat Rodprasert, Jaakko J Koskenniemi, Helena E Virtanen, Sergey Sadov, Antti Perheentupa, Helena Ollila, Jakob Albrethsen, Anna-Maria Andersson, Anders Juul, Niels E Skakkebaek, Katharina M Main, Jorma Toppari

**Affiliations:** Research Centre for Integrative Physiology and Pharmacology and Centre for Population Health Research, Institute of Biomedicine, University of Turku, Turku 20520, Finland; Research Centre for Integrative Physiology and Pharmacology and Centre for Population Health Research, Institute of Biomedicine, University of Turku, Turku 20520, Finland; Department of Pediatrics, Turku University Hospital, Turku 20520, Finland; Research Centre for Integrative Physiology and Pharmacology and Centre for Population Health Research, Institute of Biomedicine, University of Turku, Turku 20520, Finland; Research Centre for Integrative Physiology and Pharmacology and Centre for Population Health Research, Institute of Biomedicine, University of Turku, Turku 20520, Finland; Research Centre for Integrative Physiology and Pharmacology and Centre for Population Health Research, Institute of Biomedicine, University of Turku, Turku 20520, Finland; Department of Obstetrics and Gynaecology, University of Turku and Turku University Hospital, Turku 20520, Finland; Department of Public Health, University of Turku and Clinical Research Centre, Turku University Hospital, Turku 20520, Finland; Department of Growth and Reproduction, Copenhagen University Hospital—Rigshospitalet, Copenhagen DK-2100, Denmark; Centre for Research and research training in Endocrine Disruption of Male Reproduction and Child Health (EDMaRC), Copenhagen University Hospital—Rigshospitalet, Copenhagen DK-2100, Denmark; Department of Growth and Reproduction, Copenhagen University Hospital—Rigshospitalet, Copenhagen DK-2100, Denmark; Centre for Research and research training in Endocrine Disruption of Male Reproduction and Child Health (EDMaRC), Copenhagen University Hospital—Rigshospitalet, Copenhagen DK-2100, Denmark; Department of Growth and Reproduction, Copenhagen University Hospital—Rigshospitalet, Copenhagen DK-2100, Denmark; Centre for Research and research training in Endocrine Disruption of Male Reproduction and Child Health (EDMaRC), Copenhagen University Hospital—Rigshospitalet, Copenhagen DK-2100, Denmark; Department of Clinical Medicine, University of Copenhagen, Copenhagen DK-2100, Denmark; Department of Growth and Reproduction, Copenhagen University Hospital—Rigshospitalet, Copenhagen DK-2100, Denmark; Centre for Research and research training in Endocrine Disruption of Male Reproduction and Child Health (EDMaRC), Copenhagen University Hospital—Rigshospitalet, Copenhagen DK-2100, Denmark; Department of Clinical Medicine, University of Copenhagen, Copenhagen DK-2100, Denmark; Department of Growth and Reproduction, Copenhagen University Hospital—Rigshospitalet, Copenhagen DK-2100, Denmark; Centre for Research and research training in Endocrine Disruption of Male Reproduction and Child Health (EDMaRC), Copenhagen University Hospital—Rigshospitalet, Copenhagen DK-2100, Denmark; Department of Clinical Medicine, University of Copenhagen, Copenhagen DK-2100, Denmark; Research Centre for Integrative Physiology and Pharmacology and Centre for Population Health Research, Institute of Biomedicine, University of Turku, Turku 20520, Finland; Department of Pediatrics, Turku University Hospital, Turku 20520, Finland; Department of Growth and Reproduction, Copenhagen University Hospital—Rigshospitalet, Copenhagen DK-2100, Denmark; Centre for Research and research training in Endocrine Disruption of Male Reproduction and Child Health (EDMaRC), Copenhagen University Hospital—Rigshospitalet, Copenhagen DK-2100, Denmark

**Keywords:** undescended testis, testosterone, gonadotropins, Leydig cell, Sertoli cell, orchiopexy

## Abstract

**Context:**

Longitudinal data on levels of hypothalamic-pituitary-gonadal axis hormones and insulin-like growth factor I (IGF-I) during puberty in boys with a history of cryptorchidism are largely missing.

**Objective:**

We aimed to compare pubertal hormone levels between boys with a history of congenital cryptorchidism who experienced spontaneous testicular descent or underwent orchiopexy and boys without a history of cryptorchidism.

**Methods:**

This was a nested case-control study within a population-based birth cohort, with a prospective, longitudinal pubertal follow-up every 6 months (2005 to 2019). Participants were 109 Finnish boys, including boys with a history of unilateral cryptorchidism who underwent orchiopexy (n = 15), unilateral cryptorchidism who had spontaneous testicular descent (n = 15), bilateral cryptorchidism who underwent orchiopexy (n = 9), bilateral cryptorchidism who had spontaneous testicular descent (n = 7), and controls (n = 63). Serum reproductive hormone levels and testicular volumes were measured.

**Results:**

From around onset of puberty, boys with bilateral cryptorchidism who underwent orchiopexy had significantly higher follicle-stimulating hormone (FSH) and lower inhibin B levels than controls. Boys with unilateral cryptorchidism who underwent orchiopexy had significantly higher FSH than controls, whereas inhibin B levels were similar. Testosterone, luteinizing hormone, insulin-like factor 3, and IGF-I were generally similar between groups. Testicular volume of boys with unilateral or bilateral cryptorchidism who underwent orchiopexy was smaller than that of the controls from 1 year after pubertal onset (*P* < 0.05).

**Conclusion:**

Cryptorchid boys, particularly those with bilateral cryptorchidism who underwent orchiopexy, had altered levels of serum biomarkers of Sertoli cells and germ cells and smaller testicular volumes compared with controls.

Cryptorchidism (undescended testis, maldescensus testis) is a condition in which one or both testes fail to reach the normal position at the bottom of the scrotum (1). Congenital cryptorchidism is the most common congenital anomaly among newborn boys, and it has a prevalence at birth of 1.1% to 8.4% in boys with a birthweight over 2500 g (2, 3). Some testes can descend spontaneously after birth, which mostly occurs before the age of 3 to 6 months. The prevalence of cryptorchidism both at 3 and 12 months of age is approximately 1% (4, 5).

Cryptorchidism is associated with a 2- to 4-fold increased risk of testicular germ cell tumors and an increased risk of infertility, due to an impaired spermatogenesis (6–9). The consequences of cryptorchidism on testicular endocrine development during puberty are still unclear. Orchiopexy between 6 and 12 months and 18 months at the latest is nowadays the recommended treatment for cryptorchidism (10–12).

We previously reported that testicular growth in puberty was reduced among boys with a history of cryptorchidism indicating smaller Sertoli cell and germ cell populations as compared with controls (13). To date, there are few longitudinal (14) or cross-sectional studies (15, 16) on serum reproductive hormone levels during puberty in boys with a history of cryptorchidism and results are conflicting. Therefore, more data about the hypothalamic-pituitary-gonadal (HPG) hormone axis during puberty in cryptorchidism are needed. Until now, pubertal testicular function in cryptorchid boys who have spontaneous testicular descent and those who need orchiopexy is largely unknown.

Experimental and some clinical studies have suggested the role of growth hormone (GH) and insulin-like growth factor-I (IGF-I) in testicular growth (17). Receptors and binding proteins of GH or IGF-I are detected in Leydig cells, Sertoli cells, and germ cells (18). These growth factors have a role in the control of the final Sertoli cell number and testicular volume in mice (19). In humans, IGF-I levels and testicular volume increase together in early puberty (20). Infants with congenital isolated GH deficiency can have cryptorchidism (21), and recombinant human GH treatment in boys with isolated GH deficiency has been shown to increase testicular size during puberty (22). However, the role of GH and IGF-I in testicular growth in pubertal boys with a history of cryptorchidism has not been studied. This study aimed to compare the HPG hormone axis, IGF-I, and testicular volumes during puberty in boys with a history of cryptorchidism who experienced spontaneous testicular descent, boys who needed orchiopexy, and boys without a history of cryptorchidism.

## Methods

### Study Design

The design of our longitudinal pubertal follow-up has been described in detail previously (13). In brief, we have performed a prospective birth cohort study on the prevalence of cryptorchidism (4). In the cohort of 1494 prenatally recruited boys, born 1997-1999 in Turku University Hospital and examined by members of our research group, 35 had congenital cryptorchidism (4). In addition, 160 boys with congenital cryptorchidism and 24 controls born in 1997–2002 in Turku University Hospital and examined by our research team were recruited to participate in a case-control study on risk factors of congenital cryptorchidism. In the case-control study, up to 2 controls (from the prospective cohort or recruited postnatally) were selected for every boy with congenital cryptorchidism (matching criteria: date of birth ± 14 days, parity, gestational age ± 7 days, history of cigarette smoking during pregnancy [yes/no], and maternal history of diabetes mellitus [yes/no]) (4). Furthermore, every tenth boy of the remaining healthy boys in the prospective boy cohort served as controls (4). On the basis of our findings at minipuberty, there were no cases of hypogonadotropic hypogonadism in this study population. For the pubertal follow-up study, altogether 165 boys with a history of congenital cryptorchidism and 306 controls, who had participated in the prospective birth cohort study or in the case-control study and were still living close to Turku area, were invited to participate as described previously (13).

In total, 52 boys with a history of congenital cryptorchidism (31.5% of invited) and 66 of invited controls (21.6%) participated in the pubertal follow-up. One of the invited controls was not included in the previous report on testicular growth during puberty (13), because he participated only in the first visit of the pubertal follow-up. In addition, 2 boys who had participated in the prospective birth cohort wanted to participate as additional controls, because their brothers were included as official controls in the pubertal follow-up study. Therefore, the total number of controls in the pubertal follow-up was 68. Of boys with a history of cryptorchidism, 35 had had unilateral cryptorchidism (UC) and 17 had had bilateral cryptorchidism (BC). Eight subjects were excluded because of their medical history (5 UC due to monorchidism, 1 control had acquired cryptorchidism at 18 months, and 1 BC and 1 control because of precocious puberty). Furthermore, 3 controls did not consent to blood sampling. Therefore, altogether 109 subjects (63 controls, 30 UC, 16 BC) were included in the analyses. A total of 24 boys (15 UC and 9 BC) underwent orchiopexy. Spontaneous testicular descent occurred in 22 boys (15 UC and 7 BC).

None of the boys received hormonal therapy for cryptorchidism. Boys with congenital cryptorchidism were followed until 18 months of age and boys with persistent cryptorchidism were then referred to a pediatric surgeon's evaluation. The first orchiopexy was performed before puberty in all boys, with a median age of 2.0 years (range, 0.26–4.3 years), except for 1 boy who had initially had spontaneous testicular descent but was later operated for recurrent cryptorchidism at the age of 9.3 years. Three boys were operated significantly earlier (between 2 and 8 months) because of inguinal hernia and orchiopexy was performed in the same operation.

The boys were examined once every 6 months from the age of 8.5 years until there was no further testicular growth for at least 3 consecutive visits. At each visit, the medical examiner measured height and body weight and measured testicular size (both testes separately) by ruler, Prader orchidometer, and ultrasonography with the boys in the supine position. The testicular examination and ultrasonography were described in detail previously (13). Testicular volume by ultrasonography was calculated using the ellipsoid formula (π/6 × length × width^2^). The age at pubertal onset was defined as the age when one or both testes were larger than 3 mL by orchidometer at 2 consecutive visits (the age at the first visit was selected). The examinations were conducted at the Institute of Biomedicine, University of Turku in Turku, Finland from 2005 to 2019.

### Serum Hormone Measurements

Nonfasting venous blood samples were drawn from the antecubital vein. Blood samples were collected at the first visit (at the age of 8.5 years or the following visit if unsuccessful) and, then every 6 months from the age when the testicular length by ruler was > 20 mm, which is an early marker of approaching pubertal onset, until the age of full pubertal maturation (13). Blood samples were centrifuged, and serum samples were stored at −20 °C until the analysis. The median duration of serum storage before analysis was 3.5 years. The distribution of the clock time of blood sampling is shown in Table 1.

**Table 1.** Characteristics of the participants with available blood samples

	OpBC	SpBC	OpUC	SpUC	Controls
Boys at the start of study, N	9	7	15	15	63
Boys who completed the study, N	7	3	11	9	48
Testicular position at birth					
Suprascrotal or higher	9	4	13	8	—
High scrotal	—	3	2	7	—
Scrotal or retractile	—	—	—	—	63
Age at the onset of puberty (testicular size >3 mL with orchidometer), mean (SD)	11.6 years (1.0 years)	11.9 years (1.8 years)	11.4 years (0.9 years)	12.1 years (1.4 years)	11.8 years (1.0 years)
Height at the last visit, mean (SD)	176.8 cm (5.2 cm)	179.4 cm (3.6 cm)	178.0 cm (5.0 cm)	176.4 cm (7.0 cm)	179.0 cm (6.7 cm)
Time of blood sampling					
Total N	92	49	197	147	748
07:30–11:00	28	19	62	53	324
11:01–13:00	6	5	13	6	58
13:01–16:00	54	18	114	87	347
16:01–18:30	4	7	8	1	19

None of the differences between groups reached statistical significance. Abbreviations: OpBC, bilateral cryptorchidism with orchiopexy; OpUC, unilateral cryptorchidism with orchiopexy; SpBC, bilateral cryptorchidism with spontaneous testicular descent; SpUC, unilateral cryptorchidism with spontaneous testicular descent.

In total, data from 1719 examinations were available, and at least one of the hormones was measured in 1308 examinations. Serum samples from all visits were analyzed for follicle-stimulating hormone (FSH), luteinizing hormone (LH), total testosterone, inhibin B, sex hormone–binding globulin (SHBG), estradiol, IGF-I, and insulin-like growth factor-binding protein 3 (IGFBP-3). Additionally, blood samples collected at the last visit were measured for serum insulin-like factor 3 (INSL3). All samples were analyzed at the Department of Growth and Reproduction, Rigshospitalet, Copenhagen, Denmark. The technicians were blinded for the study group of the subjects and the individual succession of samples. Limits of detection, coefficients of variation, and assay details are listed in Table 2.

**Table 2.** Details of the assays used for hormonal analyses

Catalog number, RRID	Analyte	Assay (company)	Method	LOD	Intra-assay CV	Inter-assay CV
B017-201, AB_2783738	FSH	Delfia® (Perkin Elmer)	Two-sided fluoroimmunometric analysis	0.05 U/L	2.1%	2.7%
B031-101, AB_2783737	LH			0.05 U/L	3.0%	1.9%
33560, AB_2905661	Total testosterone	Access 2 (Beckman Coulter)	Chemiluminescent enzyme immunoassay	0.35 nmol/L (10 ng/dL)	4.1%	5.2%
A48617, AB_2893035	SHBG			0.33 nmol/L	4.1%	5.2%
A81303, AB_2827405	Inhibin B	Inhibin B gen II (Beckman Coulter)	Enzyme-linked immunosorbent assay (ELISA)	3 pg/mL (3 ng/L)	3.2%	10.3%
IS-3900, AB_2861357	IGF-I	Immunodiagnostic Systems	Automated chemiluminescence immunoassay	10 µg/L (10 ng/mL)	2.1%	7.2%
IS-4400, AB_2895663	IGFBP-3			80 µg/L	3.6%	13.2%
—	INSL3	In-house assay (23)	LC-MS/MS	0.06 ng/mL	<9%	—

Abbreviations: CV, coefficient of variations; LC-MS/MS, liquid chromatography–tandem mass spectrometry; LOD, limit of detection.

The Joint Ethics Committee of the University of Turku and Turku University Hospital reviewed and approved the original birth cohort study and the case-control study (7/1996, update 6/2001) and the pubertal follow-up study (12/2004). The studies were conducted according to the Helsinki II Declaration. All participants and their parents provided written informed consent.

### Statistics

The differences in normally distributed continuous variables between study groups were compared with a one-way analysis of variance (ANOVA), and by a Kruskal-Wallis test if the assumption of normal distribution was violated. Categorical variables were compared between groups with a Chi-square test.

Linear mixed-effect models for repeated measurements were used to compare the levels of reproductive hormones, IGF-I, and IGFBP-3 between boys with a history of congenital cryptorchidism who experienced spontaneous testicular descent, boys who needed orchiopexy, and controls.

Reproductive hormone levels and testicular volume were modeled on age, time of blood sampling (hours from 07:30 Am), and study group (controls, operated unilateral cryptorchidism [OpUC], spontaneously descended unilateral cryptorchidism [SpUC], operated bilateral cryptorchidism [OpBC], and spontaneously descended bilateral cryptorchidism [SpBC]) based on testicular position in our previous follow-up from birth to 18 months and a history of orchiopexy (4). The analyses were also replicated with a composite grouping (controls, unilaterally cryptorchid and bilaterally cryptorchid), and the results were very similar (data not shown).

In all models, the pubertal age was standardized for the onset of pubertal testicular growth by subtracting age from the age at the onset of puberty (ie, age 0 equals the age when testicular volume by orchidometer first exceeded 3 mL, age −0.5 equals the first visit before and age 0.5 equals the first visit after that). Covariates of models included this “pubertal age,” time of blood sampling, and study group, as well as interactions between pubertal age and time of blood sampling and study group and pubertal age. This allows for an analysis whether the differences between groups varied during the study, and controls for a potential difference in the effect of time of blood sampling during puberty. Hormone levels below the limit of detection were removed from the model. Furthermore, time points with fewer than 3 subjects in any group were excluded. Heteroscedasticity in the residuals was modeled using a variance function (power of variance) and serial correlation by first-order autoregressive or compound symmetry correlation structure. Restricted maximum likelihood was used in all final models. In the final model of FSH, the restricted maximum likelihood model converged only after exclusion of outliers. However, this model provided very similar estimates to the maximum likelihood model, which included the outliers, and the interpretation of the results did not differ between the 2 models.

Statistical modeling was done in R version 4.1.1 (24), and an R package “nlme” was used in linear mixed-effect modeling, and “emmeans” to calculate estimated marginal means, ie, model-based means. The differences in model-based means were compared using Dunnett's test. The level of significance was set at *P* < 0.05. *P* values were not corrected for multiple testing.

## Results

### Characteristics of the Study Participants

Characteristics of the participants are shown in Table 1. At the age of full pubertal maturation, boys in the operated group had smaller combined testicular volume of both testes than boys who had spontaneous testicular descent and controls. A total of 109 boys (22 boys in the spontaneous testicular descent group, 24 in the operated group, and 63 in the control group) had blood samples available for the analysis. Most blood samples were drawn between 7:30 and 11:00 or between 13:01 and 16:00.

### Associations Between Study Group and Longitudinal Serum FSH and Inhibin B Levels

**Figure 1.**
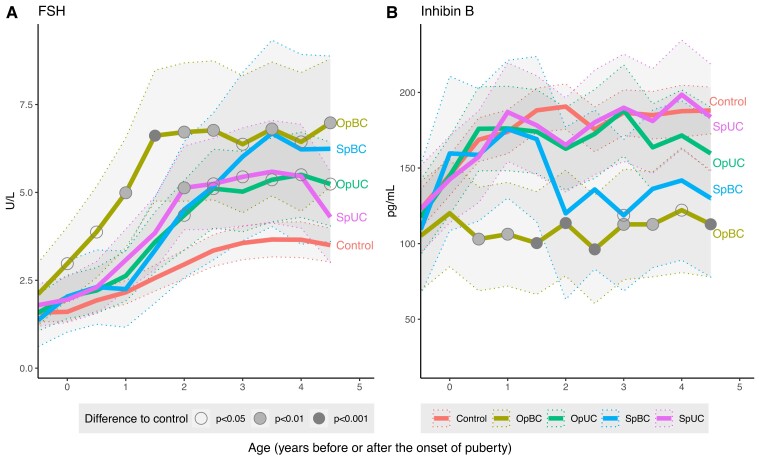
FSH and inhibin B levels in study groups based on age standardized to the onset of puberty (denoted by 0). The first time point refers to the first visit (age 8.5 years) in all subjects. The thick, continuous lines represent the modeled-based mean levels of the hormones. The thin, dashed lines and the shadowed areas represented the 95% CI for the means. Abbreviations: OpBC, bilateral cryptorchidism with a history of operation (orchiopexy); OpUC, unilateral cryptorchidism with a history of operation (orchiopexy); SpBC, bilateral cryptorchidism with spontaneous testicular descent; SpUC, unilateral cryptorchidism with spontaneous testicular descent.

The longitudinal pattern of FSH and inhibin B differed between the 5 groups (*P* = 0.02 and *P* = 0.004 for interactions between pubertal age and study group, respectively). Fig. 1 summarizes the serum FSH and inhibin B levels in UC and BC with and without a history of orchiopexy in comparison with controls and reports the level of significance of the differences. Compared with the control group, boys in the OpBC group had higher FSH and lower inhibin B levels from the onset of puberty and 0.5 years after the onset of puberty, respectively. FSH and inhibin B levels of the SpBC group seemed to follow those of controls until approximately 1.5 years after the onset of puberty, after which their FSH levels steadily increased, and inhibin B levels declined. Thereafter, curves for both hormones appeared to follow those of OpBC. However, the levels of FSH and inhibin B among boys in the SpBC group mostly did not differ from controls, probably because of the wide variability and small number of subjects in this group.

Boys in the OpUC and SpUC groups had higher FSH levels than controls at almost every time point after 2 years from the onset of puberty. Inhibin B levels in the OpUC, SpUC, and control groups were not significantly different at any time points during puberty.

### Associations Between Study Group and Longitudinal Serum LH, Testosterone, and SHBG Levels and INSL3

**Figure 2.**
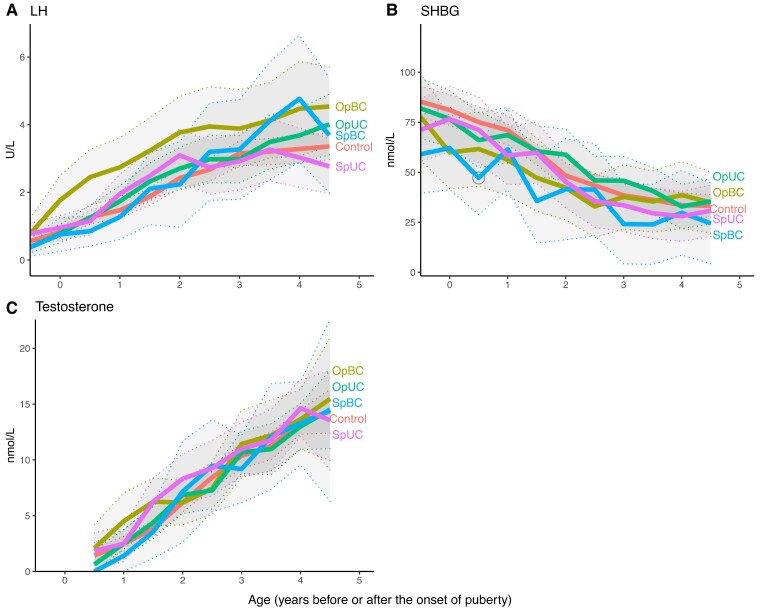
Luteinizing hormone (LH), total testosterone, and SHBG levels in study groups based on age standardized to the onset of puberty (denoted by 0). The first time point refers to the first visit (age 8.5 years) in all subjects. The thick, continuous lines represent the modeled-based mean levels of hormones. The thin, dashed lines and the shadowed areas represented the 95% confidence intervals for the means. Abbreviations: OpBC, bilateral cryptorchidism with a history of operation (orchiopexy); OpUC, unilateral cryptorchidism with a history of operation (orchiopexy); SpBC, bilateral cryptorchidism with spontaneous testicular descent; SpUC, unilateral cryptorchidism with spontaneous testicular descent. To convert total testosterone (nmol/L) to conventional unit (ng/dL), divide by 0.0347.

The longitudinal pattern of SHBG differed between the 5 groups (*P* = 0.05, Fig. 2), whereas the pattern of LH and testosterone did not (*P* = 0.21 and *P* = 0.29 for interactions between pubertal age and study group, respectively). Serum SHBG levels were lower in the SpBC group vs controls only at 0.5 year after pubertal onset. INSL3 levels at the end of puberty were not different between controls, OpBC, OpUC, SpBC, and SpUC (means ± SD: 1.5 ± 0.7 μg/L, 1.1 ± 0.4 μg/L, 1.2 ± 0.6 μg/L, 1.7 ± 1.1 μg/L, and 1.3 ± 0.4 μg/L, respectively, *P* = 0.17).

### Associations Between Study Group and Longitudinal Serum IGF-I and IGFBP-3

**Figure 3.**
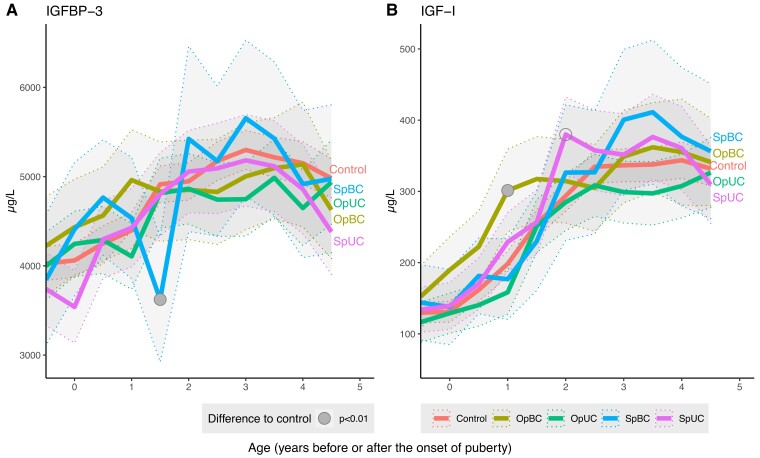
Insulin-like growth factor I (IGF-I) and insulin-like growth factor-binding protein 3 (IGFBP-3) levels in study groups based on age standardized to the onset of puberty (denoted by 0). The first time point refers to the first visit (age 8.5 years) in all subjects. The continuous lines represent the modeled-based mean levels of hormones. The thin, dashed lines and the shadowed areas represented the 95% CI for the means. Abbreviations: OpBC, bilateral cryptorchidism with a history of operation (orchiopexy); OpUC, unilateral cryptorchidism with a history of operation (orchiopexy); SpBC, bilateral cryptorchidism with spontaneous testicular descent; SpUC, unilateral cryptorchidism with spontaneous testicular descent.

Longitudinal patterns of IGF-I and IGFBP-3 (Fig. 3) differed between the 5 groups (*P* = 0.02 and *P* = 0.005 for interactions between pubertal age and study group, respectively). Although serum IGF-I levels seemed higher among boys in the OpBC group between the onset of puberty and 2 years after, the difference was significant only at 1 year after the onset of puberty. Furthermore, boys in the SpUC group had higher IGF-I than controls 2 years after the onset of puberty, whereas IGF-I levels did not differ from controls at any other time point or between controls and any other groups. Finally, IGFBP-3 levels were lower than in controls among boys in the SpBC group only at 1.5 years after the onset of puberty.

### Comparisons of the Testicular Volume Between Boys in the Spontaneous Testicular Descent, Operated, and Control Groups

**Figure 4.**
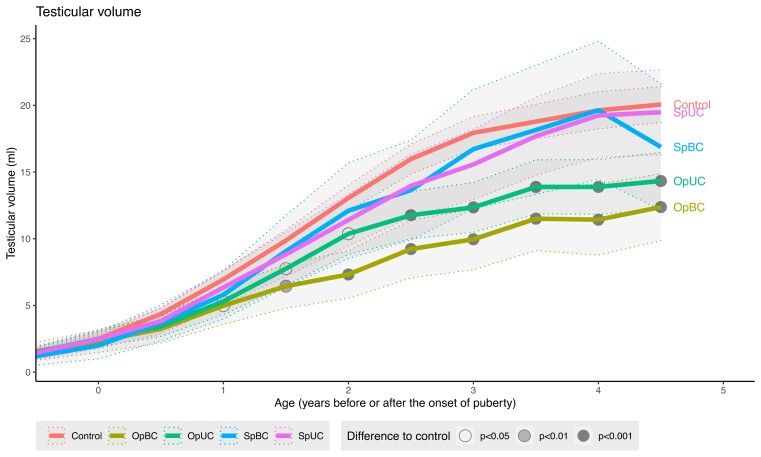
Combined testicular volume (in ultrasonography) of boys in study groups based on age standardized to the onset of puberty (denoted by 0). The first time point refers to the first visit (age 8.5 years) in all subjects. The continuous lines represent the modeled-based mean levels of volumes. The thin, dashed lines and the shadowed areas represent the 95% CI for the means. Abbreviations: OpBC, bilateral cryptorchidism with a history of operation (orchiopexy); OpUC, unilateral cryptorchidism with a history of operation (orchiopexy); SpBC, bilateral cryptorchidism with spontaneous testicular descent; SpUC, unilateral cryptorchidism with spontaneous testicular descent.

Longitudinal patterns of testicular volume differed between groups during puberty (*P* < 0.001 for interaction between pubertal age and study group). Combined testicular volume of both testes in UC and BC who underwent orchiopexy was significantly smaller than that of the controls from 1 year after pubertal onset (Fig. 4). In contrast, combined testicular volume of boys with UC and BC who had spontaneous descent did not differ from controls.

## Discussion

Our study shows that the severity of cryptorchidism plays a role for serum reproductive hormone levels during puberty, in particular with respect to FSH and inhibin B, whereas LH, testosterone, SHBG, and growth factors were not affected. In addition, our study showed that orchiopexy did not seem to normalize FSH, inhibin B, and testicular volume. Group differences of FSH and inhibin B became more pronounced as puberty progressed; therefore, normal inhibin B levels in early puberty do not always indicate normal adult Sertoli cell and germ cell number or function.

Inhibin B is a dimer that consists of α- and βB-subunits. During prepuberty, both α- and βB-subunits are produced by Sertoli cells, and therefore the inhibin B level before puberty reflects Sertoli cell number and function (25–27). After the onset of meiosis, Sertoli cells produce only the inhibin B α-subunit, and primary spermatocytes or early spermatids produce βB-subunit. Thus, following spermarche, inhibin B production is an indicator of the collaboration between germ cells and Sertoli cells, which, in turn, is also under the influence of intratesticular testosterone (26, 27).

In this light, our findings of prepubertally higher FSH and lower inhibin B levels in bilaterally cryptorchid boys who were operated suggest that they have Sertoli cell dysfunction, which may result in reduced germ cell number or function postpubertally. Although the serum FSH and inhibin B levels of the bilaterally cryptorchid boys who had spontaneous testicular descent did not differ statistically significantly from controls. It should be noted that only 7 subjects in this group started and 3 finished the study. Therefore, the comparisons between controls and this group are susceptible to random variability. Regardless of whether they were operated or not, inhibin B of unilaterally cryptorchid boys did not differ from controls and FSH differed only after the onset of puberty, indicating that their Sertoli cell function and slightly higher FSH drive may normalize their germ cell function.

Sertoli cells are the main cell population of the testis from prepuberty to early puberty and thus affect testis size (28). There were no major differences between groups during this window. However, when testosterone production increases during the course of the puberty, Sertoli cells mature and stop proliferation (28). From late puberty until adulthood, germ cells are the main contributor of testicular volume after the onset of spermatogenesis (28). Testicular volume was lower among both unilaterally and bilaterally cryptorchid boys who were operated than among controls, which points to a lower germ cell number in these boys (29). Testicular volume of unilaterally and bilaterally cryptorchid boys who had spontaneous testicular descent did not differ from controls, suggesting preserved germ cell number.

The levels of Sertoli cell and germ cell biomarkers found in our study support findings from cross-sectional studies in adult men. Men with a history of bilateral cryptorchidism had similar or higher FSH, lower serum inhibin B levels, lower sperm concentration (30, 31), and smaller testicular volume (30), indicating lower Sertoli cell and germ cell number or function than controls. Studies on men with a history of unilateral cryptorchidism were more mixed. They had comparable serum FSH, similar or lower inhibin B levels and sperm concentration, and smaller testicular volume than controls (30, 32). These results suggested preserved or some degree of reduced Sertoli cell and germ cell number or function. Our results are also in line with our previous observations that already during the postnatal activation of the HPG axis (so-called minipuberty) cryptorchid boys in general had higher FSH and lower inhibin B compared to controls and that the changes were more pronounced among boys with cryptorchidism persisting at 3 months of age (33).

In our study, LH, testosterone, and INSL3 levels were similar between cryptorchid and control groups, suggesting that Leydig cell function and/or number was preserved among boys with a history of cryptorchidism during puberty. Previous studies in adult men showed that LH and testosterone levels in men with a history of unilateral cryptorchidism did not differ from controls (30, 32). However, men with a history of bilateral cryptorchidism had higher LH but similar testosterone levels as compared with controls, suggesting compensated Leydig cell dysfunction in these men (31). We previously found that boys with cryptorchidism persisting at 3 months had decreased INSL3 levels in cord blood and elevated serum LH to INSL3 ratio at 3 months of age, which suggested decreased Leydig cell function in cryptorchid boys during the perinatal period (34).

Although IGF-I and IGFBP-3 levels were generally similar between the groups, boys in the OpBC group briefly had higher IGF-I levels than controls at early puberty. This finding might suggest a compensatory drive of testicular growth. This hypothesis is supported by some evidence showing a role of IGF-I in pubertal testicular growth (17). However, as pubertal testicular growth is impaired among boys with bilateral cryptorchidism, these boys may appear more advanced in their endocrine pubertal development than what is reflected in their testicular volume. This may explain the transient subtle differences in reproductive hormone levels between bilaterally cryptorchid and controls during early puberty when testicular size is used as a pubertal biomarker.

Boys who underwent orchiopexy appeared to have a more impaired Sertoli cell/germ cell function than those with spontaneous descent. Spontaneous, but slightly delayed, testicular descent may be seen as an extension of the physiological testicular descent, for example in preterm boys. In contrast, the need for operation may suggest an inherent testicular dysgenesis, which orchiopexy may not fully reverse. Secondly, spontaneous testicular descent primarily occurred before the age of 6 months, whereas orchiopexy was mostly performed after 6 months. Therefore, cryptorchid testis of the boys who underwent orchiopexy stayed in the undescended position longer than testis of the boys who had spontaneous testicular descent. Testis which remained in the cryptorchid position has a progressive germ cell and Sertoli cell loss (29). Previous studies showed that early orchiopexy was associated with decreased risk of infertility and development of testicular cancer (35, 36), even though some data contradicted this finding (37). Unilaterally or bilaterally cryptorchid boys who underwent orchiopexy at 3 years of age had a lower number of germ cells and Sertoli cells and smaller testicular volume as compared with boys who underwent surgery at 9 months of age (29).

The strengths of our study are the longitudinal study design and the short intervals between follow-up visits throughout puberty. In addition, the testicular position was well-documented at birth and early childhood, which allowed us to distinguish between congenital and acquired cryptorchidism which increased the homogeneity and representability of the cohort. The limitations of our study include the low number of subjects, particularly the bilaterally cryptorchid groups. This limits the statistical power to detect any differences between groups and limits the generalizability of our findings. There was variability in the time of blood sampling. We allowed blood withdrawal in the afternoon because many families were not available in the morning. However, we adjusted the analyses for the time of blood sampling in most of the statistical models to minimize the effect of variability. Finally, this study did not aim to compare the differences in testicular function in boys who had early or late orchiopexy, because the decision to operate was likely to be affected by confounders such as inguinal hernia and preterm birth.

In conclusion, hormonal changes in the FSH-inhibin B axis indicated that the main long-term consequences for testicular function in boys with congenital cryptorchidism, particularly after operation for bilateral cryptorchidism, relates to the Sertoli cell and/or germ cell development, whereas Leydig cell function is generally well-preserved during puberty.

## Data Availability

Datasets generated and analyzed during the current study are not publicly available but are available from the corresponding author on reasonable request.

## Abbreviations

BC: bilateral cryptorchidism
FSH: follicle-stimulating hormone
GH: growth hormone
HPG: hypothalamic-pituitary-gonadal
IGF-I: insulin-like growth factor I
IGFBP-3: insulin-like growth factor-binding protein 3
INSL3: insulin-like factor 3
LH: luteinizing hormone
OpBC: operated bilateral cryptorchidism
OpUC: operated unilateral cryptorchidism
SHBG: sex hormone–binding globulin
SpBC: spontaneously descended bilateral cryptorchidism
SpUC: spontaneously descended unilateral cryptorchidism
UC: unilateral cryptorchidism
